# Editorial: Understanding sex-specific issues in MS and its animal models: natural history, management and mechanisms

**DOI:** 10.3389/fneur.2024.1366126

**Published:** 2024-01-23

**Authors:** Burcu Zeydan, Manu Rangachari, Orhun H. Kantarci

**Affiliations:** ^1^Department of Radiology, Mayo Clinic, Rochester, MN, United States; ^2^Department of Neurology, Mayo Clinic, Rochester, MN, United States; ^3^Women's Health Research Center, Mayo Clinic, Rochester, MN, United States; ^4^Center for Multiple Sclerosis and Autoimmune Neurology, Mayo Clinic, Rochester, MN, United States; ^5^Axe Neurosciences, Centre de Recherche du CHU de Québec-Université Laval, Quebec City, QC, Canada; ^6^Faculté de Médecine, Université Laval, Quebec City, QC, Canada

**Keywords:** multiple sclerosis, sex, age, risk phase, progression, inflammaging, transcriptome, mitochondria

In our view, multiple sclerosis (MS) may be classified into three phases: (1) *Risk phase*, from birth to clinical/sub-clinical evidence of disease, (2) *Clinical phase*, which can be asymptomatic (radiologically isolated syndrome-RIS) or symptomatic, and relapsing or progressive, and (3) *Burnout phase*, from end of the *clinical phase* to death, primarily characterized by age-related changes in the MS background.

Significant sex differences in risk of disease, disease course, prognosis and disability are observed in MS and suggest a strong influence of sex and associated hormones on peripheral inflammatory activity, demyelination-remyelination, axonal loss, and neurodegenerative processes including astrocyte, oligodendrocyte, and microglia-mediated mechanisms (1, 2). However, our current knowledge of sex differences is mostly restricted to the *clinical phase*. Hence, better understanding of sex-specific issues in MS and its animal models is needed, which will improve sex-specific individualization of management and patient care. In this Research Topic, sex differences in MS are addressed in various ways with a deep dive into underlying mechanisms.

The article by Yusuf et al. investigates the sex and age differences in healthcare utilization in the 5 years before MS onset. The concept of events during the pre-MS diagnosis period, i.e., the “prodrome” is of interest in understanding the impact of sex during the *risk phase*. Healthcare utilization was higher in older vs. younger individuals and in males vs. females. Individuals ≥50 years were more commonly hospitalized for injury and infection, and males had higher number of antivertigo prescriptions and genitourinary-related visits. While the increased rates in older compared to younger individuals may not be surprising, the higher healthcare utilization among men relative to women deserves attention. The authors suggest that low testosterone levels may be associated with higher genitourinary-related visits in the prodromal phase in men. It is hard to assign causality, but individuals might have been selected out by having low testosterone leading to multiple simultaneous ailments including MS. These differences during the MS “prodrome” may help finetune MS risk prediction models. However, investigation beyond 5 years before MS onset is needed. More importantly, since “prodrome” is not defined by any radiological/biological evidence, some patients might have already had asymptomatic MS (i.e., RIS) and therefore utilization of imaging data for biological evidence of asymptomatic MS stratification is needed. Further, delay in earlier recognition of MS should be considered while evaluating the “prodrome” during the *risk phase* of MS.

In the next article, Alvarez-Sanchez and Dunn discuss sex differences in MS progression, which combines the concept of disability worsening and progressive MS, with a focus on biological mechanisms that overlap in these concepts. Persistence of inflammation and higher severity of neurodegeneration are some of the main drivers of sex differences in disability worsening in MS. Moreover, the peripheral immune activation leading to MS relapses may cause disability worsening when coupled with extent of injury and poor recovery with residual deficits. Chronic microglia activation and neurodegenerative mechanisms also contribute to the progressive disease process devoid of peripheral immune activation. Related to the former mechanism, the authors propose that male T cells may contribute to a more pro-inflammatory state (3). They also suggest, related to the latter mechanism, astrocytes may be more reactive in males, causing reactive gliosis (4). Males also have a higher likelihood of active rim lesions and greater iron accumulation in deep gray matter (5). Males are more susceptible to demyelination whereas females are more efficient in myelin repair (6). Altogether, females seem to have an advantage over males based on sex differences observed in cellular mechanisms associated with progression in MS. However, numerous sex differences remain unexplored in progression, particularly in humans, as many of the current observations are limited to animal models of MS.

Boziki et al. review what is known about the interaction of sex and age in MS and its animal models. They note that while males are more likely to develop progressive MS overall, females who develop MS later in life (i.e. >40 years) have an increased predisposition to developing progressive MS onset or an accelerated time to conversion to secondary progressive MS from relapsing-remitting MS (7). They link this to the intriguing concept of “inflammaging,” which refers to an increase in chronic, low-grade inflammation as people age—as compared to younger individuals with highly active immune systems. Studies suggest that in MS-affected females, overall levels of methylation are reduced relative to healthy individuals, yet this association is less apparent in males (8). Most provocatively, they discuss evidence that female microglia upregulate inflammatory markers with age; as estrogen is linked to a neuroprotective phenotype. This supports the possibility that the post-menopausal increase in female incidence of progressive MS (9) might be linked to reduced production of female sex hormones. It is also important to remember that pregnancy, which has a positive impact on MS disease course likely has direct impact on microglia functioning. This area is minimally investigated in MS.

By contrast, Itoh et al. focus on a CNS-intrinsic mechanism by which male sex may exacerbate damage. Using the experimental autoimmune encephalomyelitis (EAE) model of MS, they show cortical neuron loss is exacerbated in sick male mice relative to female counterparts. Then, using transgenic mice that express an HA-tagged *Rps22* 60S ribosomal gene (RiboTag) under the control of a neural-specific promoter, they find that there are far greater transcriptional differences between male EAE and healthy controls than between the corresponding female groups. Notably, mitochondrial dysfunction and oxidative phosphorylation pathway genes were downregulated in neurons from male EAE mice. Moving on to assess respiratory function of synaptosomes enriched for mitochondria, they showed defects in complex II function from EAE males vs females; no differences were noted between healthy males and females. Intriguingly, using the four-core genotype (FCG) mouse model, which permits the disaggregation of hormonal and chromosomal sex, the group has previously shown that the presence of XY chromosomes renders the male CNS more susceptible to neurodegeneration in the EAE model (10). Going forward, it would be interesting to assess the relative contributions of chromosomes and male sex hormones to mitochondrial dysfunction, and the impact of aging mitochondria on peripheral immune-mediated mechanisms, on cellular aging impacting repair-recovery, on activation patterns of microglia and ultimately on the induction/burnout of various processes that are associated with sex differences in MS.

In conclusion, these articles reflect a wide array of sex-specific mechanisms that operate during the lifetime of an individual. There is more work to be done to understand the chromosomal, genetic, epigenetic, environmental, hormonal, and aging related underpinnings of these mechanisms. However, studies going forward need to define the corresponding MS phenotype and phase better, as these mechanisms likely interact with or are driven by sex differently during different phases of MS. Our understanding of the impact of sex on the *clinical phase* of MS has evolved considerably but the knowledge on the *risk* and *burnout phases* of MS is at its infancy.

## Author contributions

BZ: Writing – original draft, Writing – review & editing. MR: Writing – original draft, Writing – review & editing. OK: Writing – original draft, Writing – review & editing.
